# Cognitive and cerebrovascular improvements following kinin B_1_ receptor blockade in Alzheimer’s disease mice

**DOI:** 10.1186/1742-2094-10-57

**Published:** 2013-05-04

**Authors:** Baptiste Lacoste, Xin-Kang Tong, Karim Lahjouji, Réjean Couture, Edith Hamel

**Affiliations:** 1 Laboratory of Cerebrovascular Research, Montreal Neurological Institute, McGill University, 3801 University Street, Montréal, QC, H3A 2B4, Canada; 2 Department of Physiology, Faculty of Medicine, Université de Montréal, 2900 Boulevard Edouard-Montpetit, Montréal, QC, H3T 1J4, Canada

**Keywords:** Brain kinins, Amyloid, Cerebral circulation, Neuroinflammation, Neuropeptide

## Abstract

**Background:**

Recent evidence suggests that the inducible kinin B_1_ receptor (B_1_R) contributes to pathogenic neuroinflammation induced by amyloid-beta (Aβ) peptide. The present study aims at identifying the cellular distribution and potentially detrimental role of B_1_R on cognitive and cerebrovascular functions in a mouse model of Alzheimer’s disease (AD).

**Methods:**

Transgenic mice overexpressing a mutated form of the human amyloid precursor protein (APP_Swe,Ind_, line J20) were treated with a selective and brain penetrant B_1_R antagonist (SSR240612, 10 mg/kg/day for 5 or 10 weeks) or vehicle. The impact of B_1_R blockade was measured on i) spatial learning and memory performance in the Morris water maze, ii) cerebral blood flow (CBF) responses to sensory stimulation using laser Doppler flowmetry, and iii) reactivity of isolated cerebral arteries using online videomicroscopy. Aβ burden was quantified by ELISA and immunostaining, while other AD landmarks were measured by western blot and immunohistochemistry.

**Results:**

B_1_R protein levels were increased in APP mouse hippocampus and, prominently, in reactive astrocytes surrounding Aβ plaques. In APP mice, B_1_R antagonism with SSR240612 improved spatial learning, memory and normalized protein levels of the memory-related early gene Egr-1 in the dentate gyrus of the hippocampus. B_1_R antagonism restored sensory-evoked CBF responses, endothelium-dependent dilations, and normalized cerebrovascular protein levels of endothelial nitric oxide synthase and B_2_R. In addition, SSR240612 reduced (approximately 50%) microglial, but not astroglial, activation, brain levels of soluble Aβ_1-42_, diffuse and dense-core Aβ plaques, and it increased protein levels of the Aβ brain efflux transporter lipoprotein receptor-related protein-1 in cerebral microvessels.

**Conclusion:**

These findings show a selective upregulation of astroglial B_1_R in the APP mouse brain, and the capacity of the B_1_R antagonist to abrogate amyloidosis, cerebrovascular and memory deficits. Collectively, these findings provide convincing evidence for a role of B_1_R in AD pathogenesis.

## Introduction

Alzheimer’s disease (AD) is not only characterized by cognitive and cerebrovascular deficits [1], but also by neuroinflammation that may involve the kallikrein-kinin system (KKS) [2] known to be widely distributed in brain [3]. Kinins are proinflammatory and vasoactive peptides that act through the activation of two G protein-coupled receptors, denoted as B_1_ and B_2_ (B_1_R and B_2_R). Bradykinin (BK) and kallidin (Lys-BK) are the endogenous ligands for the constitutive B_2_R whereas the C-terminal metabolites desArg^9^-BK and Lys-desArg^9^-BK are the preferential agonists of the inducible B_1_R [4]. In the brain vasculature, BK dilates most cerebral arteries [5] through activation of B_2_R and release of endothelial-derived nitric oxide (NO). BK also regulates blood–brain barrier (BBB) permeability [6]. Activation of the brain KKS occurs in hypertension [7], cerebral ischemia [8], head trauma [9] and diabetes [10], all known as important risk factors for developing AD with increased age [11].

In agreement with our preliminary studies [12,13], growing evidence also suggests a role for the KKS in AD [2], as reflected by the cleavage of high-molecular-weight kininogens observed in the cerebrospinal fluid (CSF) of AD patients [14]. Consistent with this observation, a single dose of BK infused into the rat hippocampus led to learning and memory deficits [15]. And, in rodents submitted to intracerebroventricular (i.c.v.) administration of Aβ_1–40_, BK levels were increased in the CSF [16] and BK receptors density was upregulated in memory-related brain regions such as the prefrontal cortex and hippocampus [17,18]. Moreover, pharmacological or genetic blockade of the B_1_R abrogated the cognitive deficits induced by a single i.c.v. injection of Aβ_1–40_ in rodents [18], suggesting that B_1_R could represent a target for AD therapy. Other reports indicated that B_1_R blockade protects mice from focal brain injury by controlling BBB leakage [19], a disruption evidenced in the AD brain [20].

However, a critical role for kinins in AD cognitive and cerebrovascular deficits has never been confirmed in a clinically relevant transgenic mouse model that recapitulates a wide array of AD landmarks (cerebrovascular, cognitive, neuroinflammation and amyloid pathologies), such as mice that overproduce chronically Aβ peptide through transgene expression of familial AD-related mutated human amyloid precursor protein (hAPP) [21]. Here, we sought to investigate whether B_1_R upregulation could contribute to neuronal, glial, and cerebrovascular dysfunctions in mice overexpressing the hAPP_Swe,Ind_ mutations [22] (APP mice). In addition to providing the first description of B_1_R immunoreactivity in the APP mouse forebrain, our data demonstrate that pharmacological blockade of the B_1_R counters the cerebrovascular, cognitive and anatomopathological deficits in adult APP mice with a fully developed pathology.

## Material and methods

All experimental procedures were conducted in accordance with the guidelines of the Canadian Council on Animal Care, and the protocols were approved by the Animal Care Committee at McGill University. In order to ensure the best reproducibility, experiments for each treatment cohort were conducted independently (at six-month intervals). For a rigorous interpretation of data within each cohort (for example immunohistochemistry, ELISA and western blotting), brain extracts and sections from the four experimental groups (see below) were processed simultaneously.

### Reagents and antibodies

For selective blockade of the B_1_R, the non-peptide, brain penetrant B_1_R antagonist SSR240612 [(2*R*)-2-[((3*R*)-3-(1,3-benzodioxol-5-yl)-3-{[(6-methoxy-2-naphthyl)sulfonyl]amino}propanoyl)amino]-3-(4-{[2*R*,6*S*)-2,6-dimethylpiperidinyl]methyl}phenyl)-*N*-isopropyl-*N*-methylpropanamide hydrochloride] was kindly provided by Sanofi-Aventis (Montpellier, France) [23]. SSR240612 is a stable and highly selective blocker of des-Arg^9^BK binding at B_1_R with a K_*i*_ of 0.48 nM, which was previously tested in man for neuropathic pain [24].

**Figure 1:**
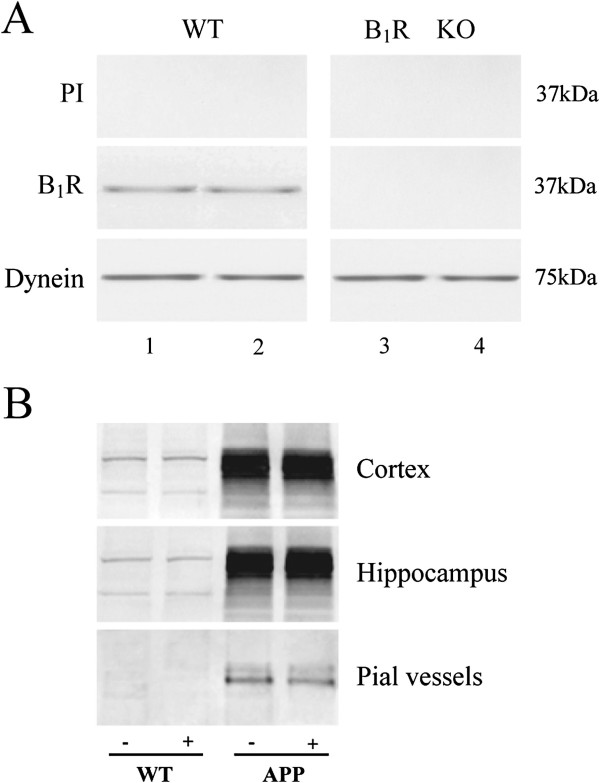
**Experimental controls. (A)** Western-blot analysis of bradykinin receptor 1 (B_1_R) pre-immune (PI) and anti-serum on kidney extracts from two wild-type (WT; 1,2) and two B_1_R knockout (KO; 3,4) mice. The anti-B_1_R antibody recognized a single band at the expected molecular weight (37 kDa), which was absent in B_1_R KO mice or in the presence of pre-immune serum. Dynein was used as standard. **(B)** Transgenic amyloid precursor protein (APP) mice featured increased expression of APP in both brain and pial vessels extracts, as measured by western blot. APP protein levels were not affected by vehicle (−) or treatment (+).

Detection of BK receptor proteins was performed by western blot and immunohistochemistry using selective anti-B_1_R and -B_2_R antibodies raised in rabbits (Biotechnology Research Institute, Montréal, QC, Canada) against a conserved amino acid sequence from B_1_R and B_2_R proteins of mouse and rat [25]. The epitopes used contained 15 amino acids localized in the C-terminal region of B_1_R (VFAGRLLKTRVLGTL) and 15 amino acids localized in the second extracellular domain of B_2_R (TIANNFDWVFGEVLC). Care was taken to avoid sequences with similarity to related mammalian proteins, including the opposite receptor. Two negative controls were run for each antibody: the pre-immune serum and the receptor-specific immunogenic peptide; both completely prevented any immunostaining. Specificity of our B_1_R antibody was further determined using mouse kidney extracts, an organ particularly rich in constitutive B_1_R [26], from wild-type (WT) and B_1_R knockout (KO) mice (kindly provided by Dr. Jean-Pierre Girolami, INSERM U1048, Université Paul Sabatier, Toulouse, France). Western blotting confirmed that the anti-B_1_R antibody recognized a single band at 37 kDa in the kidney of WT mice, which was absent from B_1_R KO mice kidney extracts (Figure 1A).

The other reagents were as follows: serotonin (5-HT) and acetylcholine (ACh) from Sigma-Aldrich (St Louis, MO, USA), BK from American Peptide (Sunnyvale, CA, USA). Antibodies were rabbit anti-beta-secretase-1 (BACE, M-83, Santa Cruz Biotechnology, Santa Cruz, CA, USA), -glial fibrillary acidic protein (GFAP, Dako, Glostrup, Denmark), -ionized calcium-binding adaptor molecule 1 (Iba1, Wako Chemicals USA, Inc., Richmond, VA, USA), -early growth response protein 1 (Egr-1 or Zif268, C-19, Santa Cruz Biotechnology,), and -matrix metallopeptidase 9 (MMP9, Millipore, Chemicon, Billerica, MA, USA), guinea pig anti-GFAP (Synaptic Systems, Goettingen, Germany), mouse anti-endothelial nitric oxide synthase (eNOS, BD Transduction Laboratories, Mississauga, ON, Canada), -amyloid-beta (Aβ)_1–16_ (6E10, Covance, Princeton, NJ, USA), -β-actin (Sigma-Aldrich), -dynein (Santa Cruz Biotechnology), rat anti-cluster of differentiation molecule 11b (CD11b, Serotec, Raleigh, NC, Canada), and goat anti-lipoprotein receptor-related protein 1 (LRP-1, N-20, Santa Cruz Biotechnology). Biotinylated, cyanin Cy2-, Cy3- or Cy5- and horseradish peroxidase-conjugated secondary antibodies were from Vector Laboratories (Burlingame, CA, USA) and Jackson Laboratories (West Grove, PA, USA), respectively. Avidin-biotin complex (ABC), 3,3′-diaminobenzidine (DAB) and slate gray (SG) reagent kits were from Vector Laboratories. ECL Plus kit for enhanced chemiluminescence was from Amersham (Mississauga, ON, Canada).

### Animals and treatment

We used heterozygous transgenic adult C57BL/6 mice (10 months old) that express the human amyloid precursor protein (hAPP) carrying the Swedish (K670N, M671L) and Indiana (V717F) familial AD mutations directed by the platelet-derived growth factor (PDGF) β-chain promoter (APP mice, J20 line) [22] and age-matched WT littermates, with approximately equal numbers of females and males. None of the parameters measured in the present study were affected by gender. We selected the J20 mice because at 10 months of age they display the full spectrum of cerebrovascular, cognitive, neuroinflammation and amyloid pathologies [22,27,28], hence allowing therapeutic rescue rather than preventive intervention, which we believe has high relevance for AD patients. Two cohorts (n ≥48 each) were divided in four groups (WT-vehicle, WT-treated, APP-vehicle, APP-treated) and treated with SSR240612 for periods of 5 or 10 weeks. Due to compound solubility and minipump limitations, the maximal administrable dose was 10 mg/kg/day. As promising, yet incomplete beneficial effects were observed after 5 weeks of treatment, a second cohort received SSR240612 during 10 weeks. SSR240612 was diluted at 215 mM in 40% DMSO and 60% sterile saline (NaCl) added in this sequence. SSR240612 (10 mg/kg/day) and vehicle (40% DMSO, 60% NaCl) were delivered (0.11 μl/hr, 5 weeks) through osmotic minipumps (model 1004, Alzet™, Durect Corporation, Cupertino, CA, USA), which were implanted subcutaneously under isoflurane anesthesia. For the treatment of 10 weeks, minipumps were removed after 5 weeks and replaced by new ones for an additional 5-week period. Treatment did not affect APP transgene expression in brain or vascular tissues (Figure 1B).

### Morris water maze

The ability of mice to learn and remember the location of a platform in a predefined (target) quadrant in a circular pool filled with opaque water (17°C) using visuospatial cues was tested during eight consecutive days (three days of visible platform training followed by five days of hidden platform trials), as described elsewhere [29]. Platform location and visual cues distribution were altered between the visible and hidden platform testings. Twenty-four hours after the last hidden platform trial, on day 9, mice were submitted to a 60-sec probe trial (platform removed). Visual acuity and locomotor ability were comparable between all groups, as assessed during the visible platform testing. All experiments were started at the same time every day. Daily escape latencies to the platform, percentage of time spent and distance traveled in the target quadrant during the probe trial, along with swim speed, were collected with the 2020 Plus tracking system and analyzed with the Water 2020 software (HVS Image, Buckingham, UK).

### Laser Doppler flowmetry

Laser Doppler flowmetry measurements (Transonic Systems Inc., Ithica, NY, USA) of evoked cerebral blood flow (CBF) in response to sensory stimulation were carried out one week following the Morris water maze in anesthetized mice (n = 4 to 6, ketamine 80 mg/kg intraperitoneally; Wyeth, St-Laurent, QC, Canada) fixed in a stereotaxic frame [28]. CBF was recorded over the contralateral somatosensory cortex before, during and after unilateral stimulation of the right whiskers (20 sec at 8 to 10 Hz). Six recordings were acquired every 30 to 40 sec and averaged for each mouse. The entire procedure lasted less than 30 min, a time window when all physiological parameters remain stable [30]. Cortical CBF changes were expressed as percentage increase relative to baseline.

### Vascular and brain tissue collection

Mice were killed by cervical dislocation and middle cerebral artery (MCA) segments immediately tested in vascular reactivity studies. For immunohistochemistry (IHC), western blot (WB) and ELISA studies, mice were exsanguinated by intracardiac perfusion of sterile 0.9% NaCl under deep sodium pentobarbital anesthesia. Vessels of the circle of Willis and their branches free of pial membrane along with cortex and hippocampus of one hemibrain were collected, snap-frozen on dry ice and stored (−80°C). The other hemibrain was fixed by overnight immersion in 4% paraformaldehyde (PFA) in 0.1 M phosphate-buffered saline (pH 7.4), cryoprotected, frozen in isopentane (−45°C) and stored (−80°C) until cutting into 25 μm-thick free-floating coronal sections using a freezing microtome.

### Vascular reactivity

In order to assess the impact of B_1_R blockade on the reactivity of cerebral vessels, isolated, pressurized and submaximally precontracted (5-HT, 2.10^-7^ M) MCA segments (diameter 40 to 70 μm) from WT and APP mice were tested for dilatation to ACh (10^-10^ to 10^-5^ M) and BK (10^-10^ to 10^-5^ M) using online videomicroscopy [31]. Percentage changes in vessel diameter from pre-constricted tone were plotted as a function of agonist concentration. The maximal response (EAmax) and the concentration eliciting half of EAmax (EC_50_ value, or pD_2_ = −[log EC_50_]), were generated by the GraphPad Prism software (version 4, San Diego, CA, USA) and used to evaluate agonist efficacy and potency, respectively.

### Immuno- and histochemical staining

Sections were pretreated with 3% H_2_O_2_ (20 min) and incubated overnight at room temperature (RT) with either rabbit anti-B_1_R (1:1500) or -Egr-1 (1:250) antibodies diluted in a blocking buffer, followed by biotinylated anti-rabbit IgGs and the ABC kit; labeling was revealed with 0.05% DAB (B_1_R) or SG (Egr-1). To study basal Egr-1 expression levels and avoid task-induced changes [32], Egr-1 immunohistochemistry was done on animals sacrificed three days post-water maze. For detection of dense core amyloid plaques, sections from APP mice were stained with 1% thioflavin-S (8 min). Sections from all groups were incubated with rabbit anti-GFAP (1:400), -Iba1 (1:300), mouse anti-Aβ_1–16_ (1:1000), or goat anti-LRP-1 (1:250), followed by species-specific Cy2- (GFAP and Iba1) or Cy3- (LRP-1) conjugated secondary antibodies (1:300) for the detection of activated astrocytes, microglia, diffuse and dense-core Aβ plaques, or LRP-1, respectively. Sections were observed under a Leitz Aristoplan microscope using bright field or an FITC filter and epifluorescence (Leica, Montréal, QC, Canada) and digital pictures were acquired with a digital camera (Coolpix 4500; Nikon, Tokyo, Japan). For double immunofluorescence, sections were simultaneously incubated with a rabbit anti-B_1_R antibody and either a guinea pig anti-GFAP, rat anti-CD11b, or mouse anti-Aβ_1–16_ antibody, followed by donkey anti-rabbit Cy3- and species-specific Cy2-conjugated IgGs. Triple immunolabeling was also performed by co-incubating sections with rabbit anti-B_1_R (with Cy5 secondary), rat anti-CD11b (Cy2) and mouse anti-Aβ_1–16_ (Cy3) antibodies. Sections were observed and images acquired under a Zeiss LSM 510 laser scanning confocal microscope (Carl Zeiss Ltd., Toronto, ON, Canada) equipped with appropriate filters.

### Staining quantification

Digital images (two to three sections/mouse, n = 4 to 6 mice) taken under the same conditions were analyzed with MetaMorph (6.1r3, Universal Imaging, Downingtown, PA, USA). The areas of interest (somatosensory/cingulate cortex, dorsal hippocampus) containing thioflavin S-, 6E10-, GFAP-, Iba1- and LRP-1-positive elements were manually outlined in MetaMorph on low-power digital pictures (as exemplified in Figure 1A by dashed lines), whereas high-power images of the hippocampus were used for quantification of Egr-1 immunostaining. For microglial proliferation, the numbers of Iba-1-positive cell bodies were manually counted on digital pictures (two pictures from cortex, one from hippocampus including the dentate gyrus (DG), from three immunostained sections per mouse). The area occupied by Iba1- and GFAP-positive cells was also quantified and expressed as a percentage of surface occupied by labeling within the delineated areas of interest. Since clusters of activated microglial emit a stronger immunofluorescence signal, the maximal intensity of Iba-1 labeling was measured by dividing the maximal gray value of staining by the total gray value of the delineated area. The area occupied by thioflavin S- and 6E10-positive plaques, as well as by LRP-1-positive cells was also measured, together with the intensity (total gray value) of LRP-1 immunofluorescence. For better LRP-1 staining illustration, colors on micrographs were inverted and desaturated. Quantification of Egr-1 immunoreactivity in the hippocampus included both the total gray value in CA1-CA2 areas and the number of positive nuclei in the DG.

**Figure 2:**
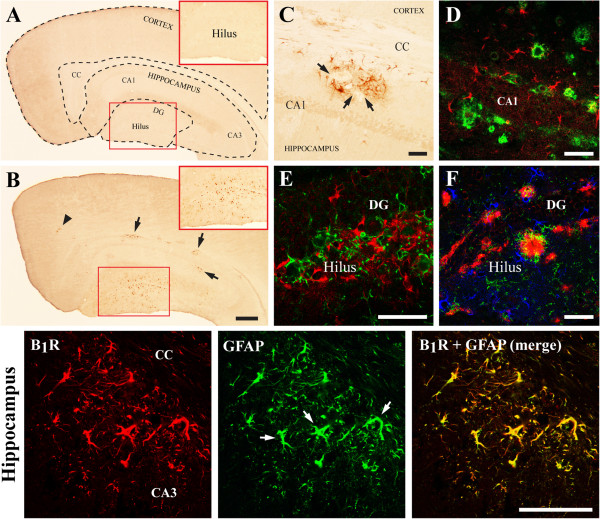
**Distribution of B**_**1**_**R in WT and APP mouse forebrain: co-localization with reactive astrocytes. (A,B)** Single B_1_R immunohistochemical labeling (revealed with DAB, brown precipitate) in the somatosensory cortex and dorsal hippocampus of WT **(A)** and APP **(B)** mice. B_1_R were upregulated in the APP mouse brain (insets: high magnification of dentate gyrus (DG)). **(C-F)** High magnification of B_1_R immunolabeling in APP mice hippocampus. **(C)** Single B_1_R immunoreactive cells (DAB staining) surrounding unlabeled parenchymal zones (arrows). **(D)** Double immunofluorescence of B_1_R-positive cells (red) intermingled around small Aβ plaques (green). (E) Double immunofluorescence of B_1_R (red) and CD11b (green) showed that they label distinct cellular elements. **(F)** Triple immunofluorescence of B_1_R (blue), Aβ (red) and CD11b (green) showed that B_1_R-positive cells surround the outside rim of Aβ plaques, and are distinct from CD11b-positive microglial cells. Lower panel: double immunofluorescence of B_1_R (red) and GFAP (green) in hippocampus of APP mice confirmed that B_1_R immunoreactivity is predominantly localized in astroglial cells. DG, dentate gyrus; CC, corpus callosum. Scale bars: 100 μm, except in B: 300 μm. N = 3 to 8/group. Aβ, amyloid-beta; APP, amyloid precursor protein; B_1_R, bradykinin receptor 1; CD11b, cluster of differentiation molecule 11b; GFAP, glial fibrillary acidic protein; WT, wild-type.

### Enzyme-linked immunosorbent assay (ELISA)

Levels of soluble Aβ_1–40_ and Aβ_1–42_ were measured in homogenized hemibrains from APP mice (n = 4 to 5) using ELISA kits (BioSource International, Camarillo, CA, USA), as described before [28]. Data were collected as optical density values in the tissue supernatants and expressed as a percentage of untreated APP mice.

### Western blot

For protein quantification (n = 5 to 6 mice/group), vessels were homogenized in Laemmli buffer and cortex and hippocampus in a lysis buffer [31]. In brief, extracts were protein assayed, loaded (5 to 50 μg) in 10% SDS-polyacrylamide gels, separated by electrophoresis and transferred to nitrocellulose membranes. Membranes were incubated (1 h, RT) in a blocking buffer containing 5% skim milk and then (overnight, 4°C) with either rabbit anti-B_1_R (1:500), -B_2_R (1:500), -BACE (1:1000), -MMP9 (1:1000), or mouse anti-Aβ_1-16_ (1:1000), -eNOS (1:1000), -β-actin (1:10000), or -dynein (1:25000). Membranes were further incubated (1 h, RT) with horseradish peroxidase-conjugated secondary antibodies (1:2000) and proteins visualized by chemiluminescence (ECL Plus kit) using a phosphorImager (Scanner STORM 860; GE Healthcare, Piscataway, NJ, USA), followed by densitometric quantification (ImageQuant 5.0, Molecular Dynamics, Sunnyvale, CA, USA).

### Statistical analysis

All data are means ± SEM and were analyzed with GraphPad 4 (San Diego, CA, USA) or Statistica 10 (StatSoft, Tulsa, OK, USA) software. Two-group comparisons (effects of treatment on amyloidosis) were analyzed by unpaired Student’s *t* tests. Four-group comparisons were analyzed using two-way analysis of variance (ANOVA, genotype and treatment as factors) followed by a Newman-Keuls post hoc test when the interaction or at least one factor was significant. *P* <0.05 was considered significant. Except for vascular reactivity, anatomical and western blot studies, experiments were conducted blind to the mouse identity. The relationships between amyloid burden and cognitive performance or LRP-1 immunoreactivity were assessed by plotting the averages of Aβ plaque load (percentage area) from thioflavin-S and 6E10 stainings with those of the cognitive score (percentage of time and percentage of distance spent in the target quadrant) or LRP-1 immunostaining (percentage of intensity or percentage of occupied area) in the cortex, hippocampus and DG in five non-treated and five treated APP mice from the 10-week treatment cohort.

## Results

### Upregulation of B_1_R in the APP mouse brain

Since localization and expression of BK receptors in the rodent brain has been described by autoradiography of radioligand binding sites and by western blot [9,17,18], we first investigated the immunohistochemical distribution of the B_1_R protein in WT and APP mice using highly selective primary antibodies. B_1_R immunoreactivity was very low to absent in the cortex and hippocampus of WT mice (Figure 2A), with only a faint, barely detectable staining in the DG (Figure 2A, inset). In APP mice, B_1_R immunostaining was increased in the hippocampus (Figure 2B, arrows), primarily in the DG that displayed abundant immunopositive cells in the hilus region (Figure 2B, inset), whereas only few B_1_R-positive cells were found in cortex (Figure 2B, arrowhead). In APP hippocampus (Figure 2B,C), B_1_R-positive clusters contained glia-shaped cells with hypertrophied processes surrounding empty parenchymal zones characteristic of amyloid deposition, as confirmed in high-power micrographs of double immunofluorescence for B_1_R and Aβ_1-16_ (Figure 2D; see also 2F). The upregulated B_1_R immunoreactivity did not co-localize with the CD11b marker for activated microglia (Figure 2E,F), but with the typical astroglial GFAP marker (Figure 2, bottom). B_1_R-positive astrocytes in APP mice displayed enhanced GFAP immunostaining, hypertrophic processes and soma (Figure 2, bottom, white arrows). No B_1_R-positive blood vessels were detected in pia or parenchyma of the mouse brain, in accordance with B_1_R not mediating BK-mediated vasomotor responses [33].

**Figure 3:**
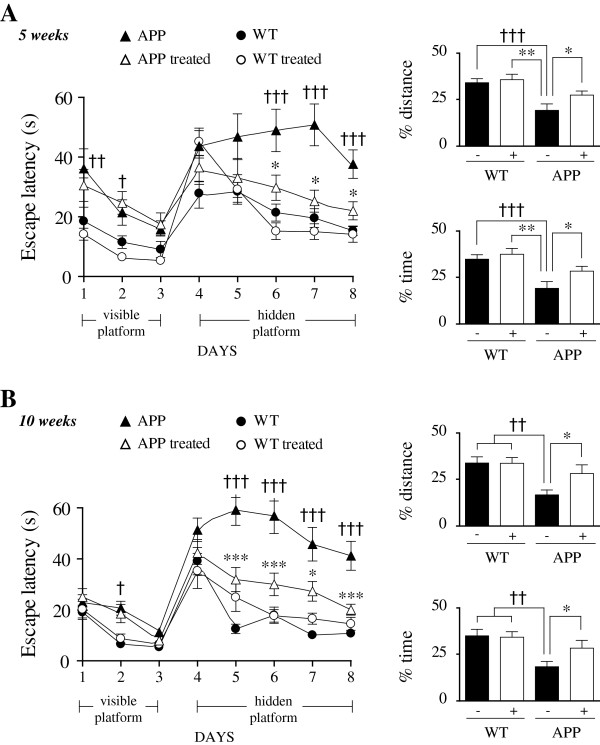
**SSR240612 treatment improved spatial learning and memory in APP mice after 5 (A) and 10 weeks (B) of treatment.** In the Morris water maze, APP mice (▲) displayed impaired learning in the hidden-platform testing (left), and memory deficit in the probe trial (platform removed) performed on day 9 (right) compared to both non-treated WT (●) and treated WT (○) controls. SSR240612 partly rescued these deficits in treated APP mice (Δ) that did not statistically differ from WT control groups. SSR240612 had no effect in WT. APP mice did not feature motor or visual deficits as shown by their ability to reach the visible platform as effectively as WT by day 3. Error bars represent SEM. *P <0.05; **, ††*P* <0.01; ***, †††*P* <0.001. Two-way ANOVA followed by Newman-Keuls post hoc test (†: APP vs. WT, *: APP vs. APP treated). N = 10 to 15/group. APP, amyloid precursor protein; WT, wild-type.

### Improvement of learning and memory following B_1_R blockade in APP mice

In order to investigate whether B_1_R blockade could alter behavioral outcome in APP mice, cognitive performances were assessed in the Morris water maze paradigm in our two cohorts of mice. Although APP mice were slightly slower than WT mice in reaching the visible platform at specific time points, they had no visual or motor deficit and all groups performed similarly on day 3 (Figure 3A,B). In contrast, both learning and memory were significantly impaired in APP mice compared with WT littermates. APP mice displayed increased latencies to reach the hidden platforms (Figure 3A,B, left) and reduced time spent and distance traveled in the target quadrant (−50%; *P* <0.01) during the probe trial (Figure 3A,B, right) although swim speeds were similar among all groups (data not shown). Both 5 and 10 weeks of SSR240612 treatment greatly improved learning and memory performances in APP mice, without any effect on WT mice. Recovery was comparable after 5 or 10 weeks of drug delivery.

**Figure 4:**
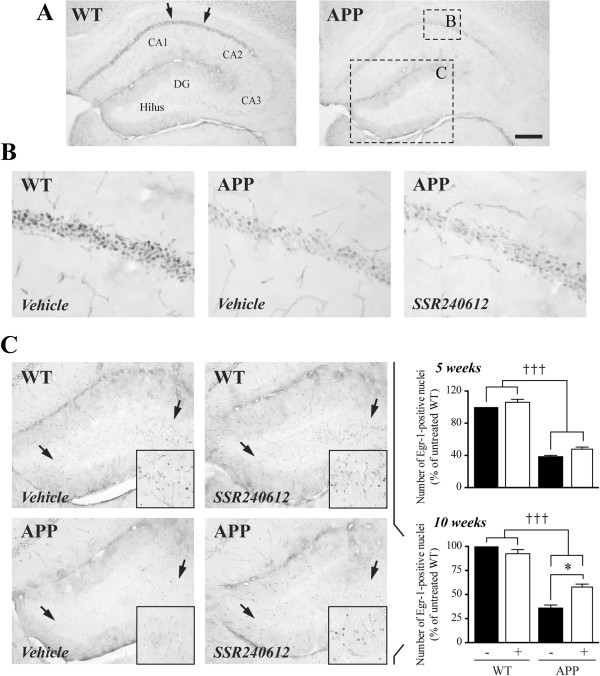
**Decreased basal Egr-1 protein levels in APP mice hippocampus are partially restored in the dentate gyrus after 10 weeks of SSR240612 delivery. (A)** Immunohistochemistry in the dorsal hippocampus revealed a drastic reduction in Egr-1 staining intensity (total gray value) in the CA1-CA2 fields **(B)**, and in the number of Egr-1-positive nuclei in dentate gyrus DG, **(C)** of APP mice. SSR240612 treatment had no effect on Egr-1 expression in CA1 **(B)**. **(C)** In contrast, following 10 weeks of delivery, SSR240612 partially countered Egr-1 reduction in the DG of APP mice. Quantified areas are delineated in A (arrows in WT; dashed boxes in APP) and magnified in B and C. See insets for higher magnifications. Scale bar: 300 μm. *P <0.05; ††*P* <0.01; †††*P* <0.001 (†: vs. WT; *: non-treated APP vs. treated APP). Two-way ANOVA followed by Newman-Keuls post hoc test. N = 5/group. APP, amyloid precursor protein; Egr-1, early growth response protein 1; WT, wild-type.

### Normalization of Egr-1 protein levels in the DG of the hippocampus in SSR240612-treated APP mice

We then studied Egr-1 (Zif268) protein levels in hippocampus, a transcription factor related to synaptic activity [34], and required for memory induction and consolidation [35]. Basal expression of Egr-1 is reduced in APP mice [30,36], and upregulated in adult APP mice with pharmacologically restored memory [30]. We confirmed a drastic reduction (50 to 70%, *P* <0.001) of Egr-1 staining intensity in the CA1-CA2 region (Figure 4A,B) of the hippocampus in APP mice. However, despite significant improvement in memory after SSR240612, no beneficial effect of treatment was evidenced on Egr-1 expression in this region. In contrast, in addition to a higher Egr-1 staining intensity readily detectable in the hilus of the DG of treated compared to untreated APP mice (Figure 4C), a partial but significant recovery (+25%; *P* <0.05) in the reduced number (−60%; *P* <0.001) of Egr-1-positive nuclei was also observed following 10, but not 5 weeks of SSR240612 treatment (Figure 4C).

**Figure 5:**
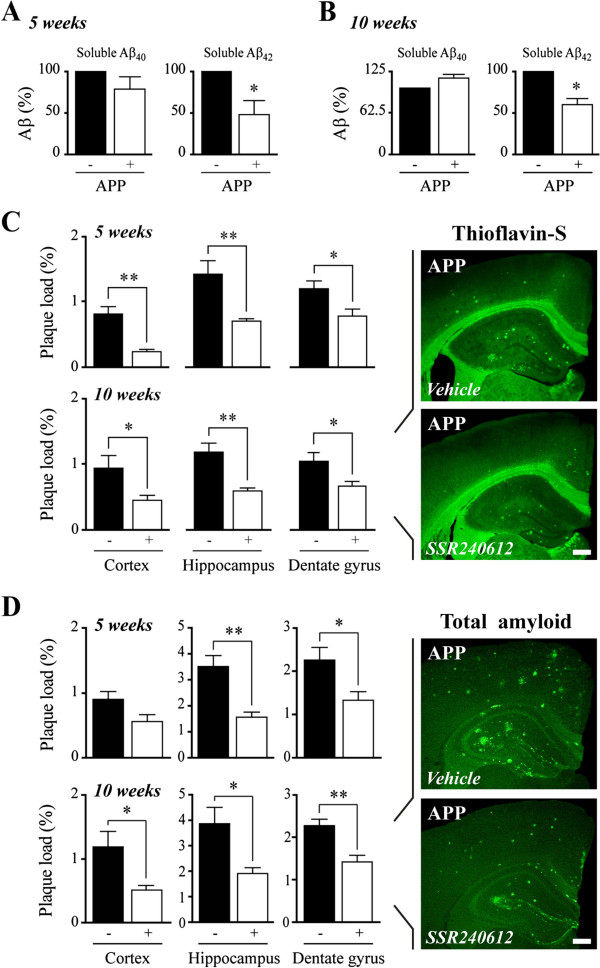
**SSR240612 reduced soluble Aβ**_**1–42 **_**in hemibrains and Aβ plaque load in cortex and hippocampus. (A,B)** Levels of soluble Aβ_1–42_ were significantly reduced in the brain of APP mice after 5 **(A)** and 10 weeks **(B)** of B_1_R blockade, as observed by ELISA. **(C,D)** Following SSR240612 treatment, thioflavin-S staining **(C)** and 6E10 immunohistochemistry **(D)** revealed a significant reduction in the surface area occupied by mature and diffuse Aβ plaques (plaque load) in both cortex and hippocampus (including the dentate gyrus). Error bars represent SEM. *P <0.05; **P <0.01, Student’s *t* test. N = 4 to 6/group. Aβ, amyloid-beta; APP, amyloid precursor protein; B_1_R, bradykinin receptor 1.

### B_1_R blockade reduced soluble Aβ_1–42_ species and amyloidosis

**Figure 6:**
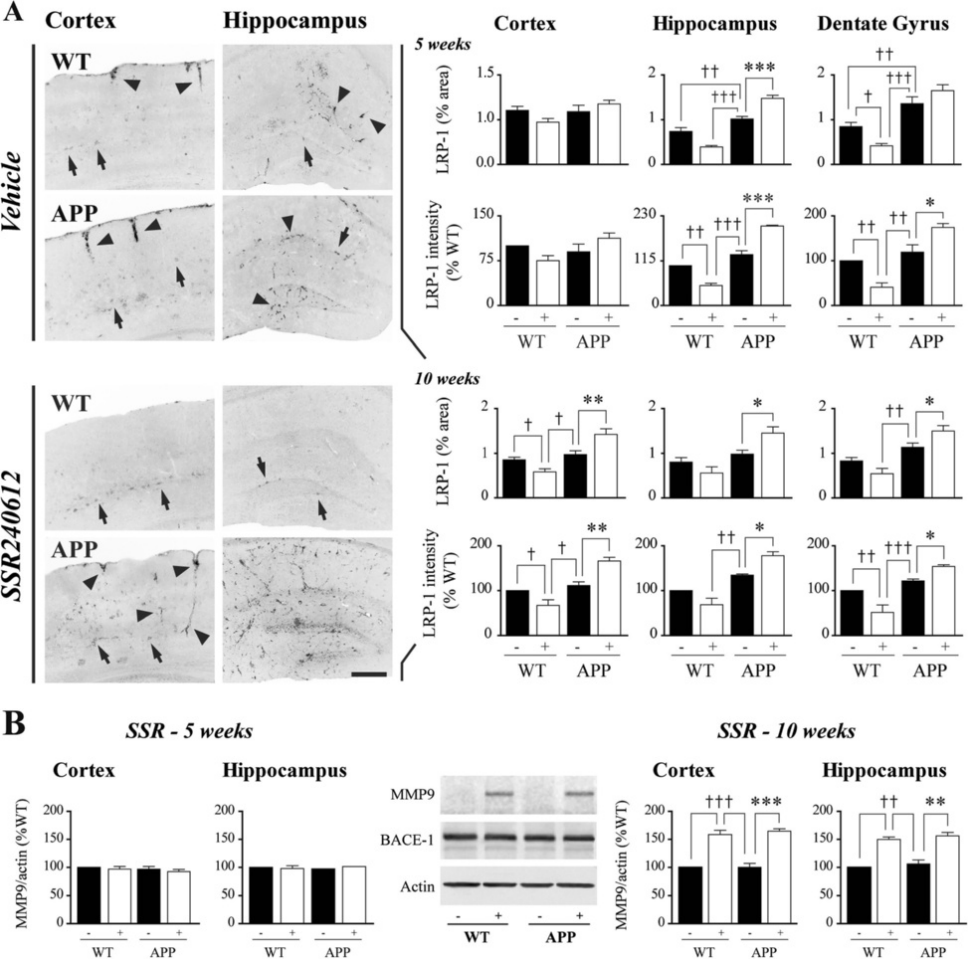
**Effects of B**_**1**_**R blockade on brain and vascular LRP-1 and brain MMP9. (A)** SSR240612 increased LRP-1 protein levels in the APP mouse brain. Vascular (arrowheads), but not neuronal (arrows), lipoprotein receptor-related protein 1 (LRP-1) immunolabeling (left) was altered by genotype and/or treatment. LRP-1 immunostaining associated to brain vessels was decreased in treated WT mice and slightly increased in untreated APP mice. SSR240612 further increased LRP-1 vascular immunoreactivity in APP mice, in the hippocampus after 5, and in both cortex and hippocampus after 10 weeks of drug administration. Scale bar: 300 μm. **(B)** Additionally, after 10 but not 5 weeks of drug administration, the protein levels of MMP9 were increased by 50 to 60% in the brain of treated animals. Blots images illustrate the hippocampus from the 10 weeks of treatment cohort. Error bars represent SEM. *, †*P* <0.05; **, ††*P* <0.01; ***, †††*P* <0.001 (†: vs. WT; *: non-treated APP vs. treated APP). Two-way ANOVA followed by Newman-Keuls post hoc test. N = 5 to 6/group. APP, amyloid precursor protein; B_1_R, bradykinin receptor 1; MMP9, matrix metallopeptidase 9; WT, wild-type.

As expected [22], transgenic APP mice featured high brain levels of soluble Aβ peptide, as measured by ELISA (Aβ_1-40_ and Aβ_1-42,_ Figure 5A,B), and of aggregated and deposited Aβ species as measured by thioflavin-S staining (dense-core Aβ plaques, Figure 5C) and 6E10 immunohistochemistry (total amyloid, Figure 5D). Following short- and long-term SSR240612 administration, levels of soluble Aβ_1-42_ species were selectively decreased in hemibrains (Figure 5A,B), and mature and diffuse Aβ plaque loads were significantly reduced in the somatosensory/cingulate cortex and dorsal hippocampus (Figure 5C,D). Both the 5- and 10-week treatments exerted reducing effects on amyloidosis, but the total Aβ plaque load in neocortex was significantly decreased only after the longer treatment (Figure 5D).

### B_1_R blockade, lipoprotein receptor-related protein-1 (LRP-1) and matrix metallopeptidase 9 (MMP9)

Since brain Aβ homeostasis is highly regulated by Aβ clearance at the BBB by LRP-1 [37,38], we investigated whether SSR240612 treatment could impact on this Aβ efflux system. WT and APP mice showed few LRP-1-immunoreactive neurons in cortex and hippocampus, with immunoreactive vessels being present along the cortical surface and throughout the hippocampus (Figure 6A). SSR240612 treatment decreased LRP-1 immunoreactivity in brain parenchyma and microvessels of treated WT mice. In contrast, 5 and 10 weeks of SSR240612 treatment significantly increased (approximately 30 to 40%) LRP-1-labeled area and intensity in APP mice depending on the brain region (Figure 6A). Neuronal LRP-1, in contrast, remained unchanged after treatment, compatible with neuronal LRP-1 expression being independent of amyloidosis in AD mice [39]. As MMP9 is involved in both BBB integrity and Aβ clearance [40-43], its protein levels were also measured. Following 10 but not 5 weeks of drug administration, a 50 to 60% increase (*P* <0.01) in MMP9 protein levels was evidenced in the cortex and hippocampus of WT and APP mice (Figure 6B), whereas those of the Aβ-generating enzyme BACE-1 were not altered (Figure 6B), the latter supporting that SSR240612 acted beyond Aβ synthesis.

### SSR240612 reduced microglial, but not astroglial, activation in APP mice

**Figure 7:**
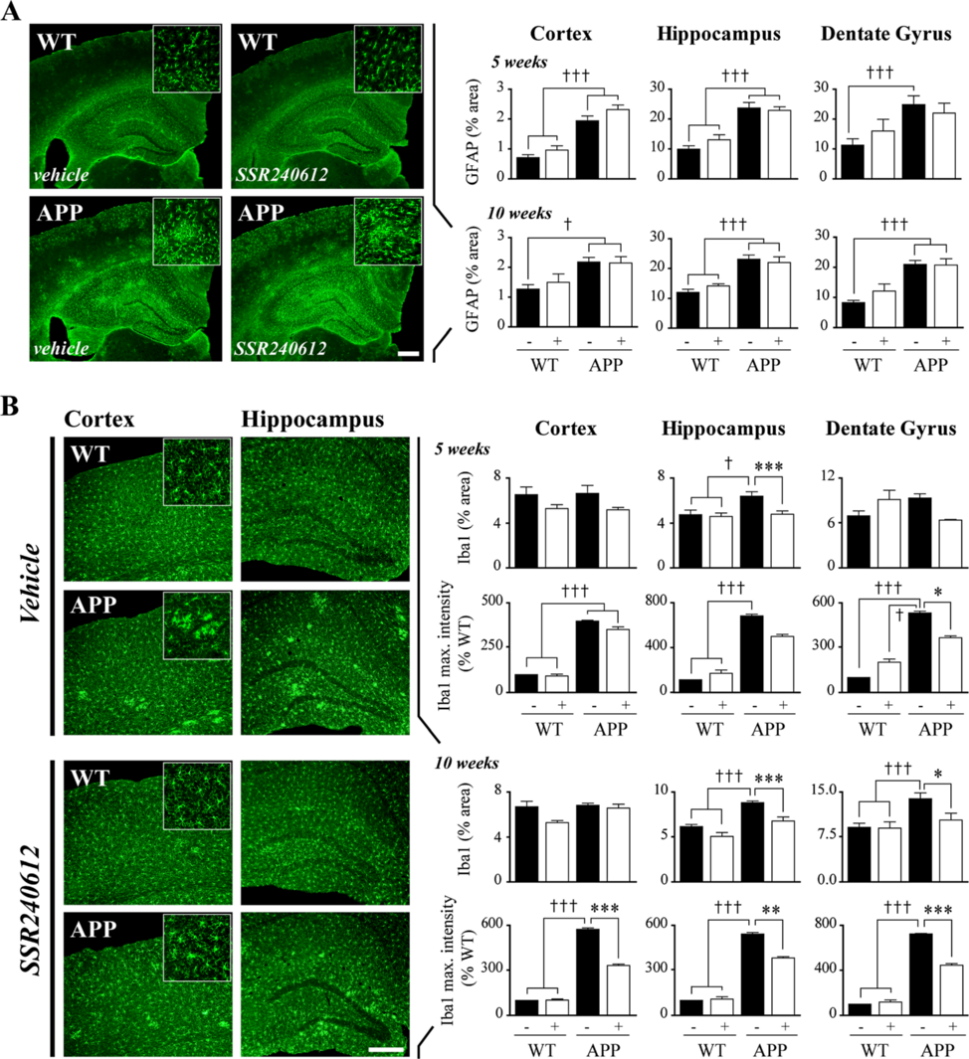
**SSR240612 reduced microglial, but not astroglial, activation in the APP mouse brain. (A)** As observed with glial fibrillary acidic protein (GFAP) immunolabeling (left), 5 and 10 weeks of treatment did not reduce astrocytosis in APP mice. **(B)** Following immunolabeling of microglial marker Iba1, SSR240612 treatments (10 weeks, left, compared to vehicle-treated mice) significantly reduced microglial activation (percentage of positive area) and aggregation (maximal labeling intensity). High magnifications are shown in insets in A and B. After 5 weeks of treatment, the effects were barely significant in hippocampus including the dentate gyrus (DG). Scale bars: 300 μm. Error bars represent SEM. *, †*P* <0.05; **, ††*P* <0.01; ***, †††*P* <0.001 (†: vs. WT; *: non-treated APP vs. treated APP). Two-way ANOVA followed by Newman-Keuls post hoc test. N = 5 to 6/group. APP, amyloid precursor protein; Iba1, ionized calcium-binding adaptor molecule 1; WT, wild-type.

Knowing that neuroinflammation mediated by astrocytes and microglia is induced in AD [44], as replicated in the brain of APP mice by the enhanced GFAP (astrocytes, Figure 7A) and Iba1 (microglia, Figure 7B) immunofluorescence, we sought to investigate whether the SSR240612-induced decrease in amyloidosis would be accompanied by a reduction in activated glial cells. Upregulated GFAP immunostaining in APP mice occurred primarily as patchy islands (Figure 7A, insets) reminiscent of Aβ deposits in neocortex and hippocampus, but SSR240612 treatment (5 and 10 weeks) did not lessen the extent of astroglial activation (Figure 7A). In contrast, the increase in microglial Iba1-positive area and, mainly, Iba1 staining intensity in APP brain were significantly reduced following SSR240612 treatment (Figure 7B). The patchy areas associated with plaques (Figure 7B, insets) were much less apparent in all regions after 10 weeks of drug delivery. Hippocampus, including the DG, was more responsive to treatment than cortical areas. In contrast, microglial proliferation was not affected by genotype or by treatment, as evidenced by the unchanged Iba1-positive cells number in all conditions (Table 1).

**Table 1:** Counts of Iba1-positive cell bodies in the cortex and hippocampus from the two treatment cohorts

**SSR 5 weeks**	**WT (n = 5)**	**WT-SSR (n = 5)**	**APP (n = 5)**	**APP-SSR (n = 4)**
Cortex	542.5 ± 19.3	546.1 ± 46.5	536.3 ± 29.5	497.2 ± 8.8
Hippocampus	532.4 ± 36.8	553.9 ± 57.3	524.2 ± 37.1	518.2 ± 16.4
Dentate gyrus	418.1 ± 50.6	464.5 ± 62.9	439.6 ± 29.1	433.0 ± 60.8
**SSR 10 weeks**	**WT (n = 5)**	**WT-SSR (n = 5)**	**APP (n = 5)**	**APP-SSR (n = 5)**
Cortex	529.9 ± 27.1	557.8 ± 18.1	549.5 ± 11.9	576.4 ± 50.9
Hippocampus	608.2 ± 15.4	624.8 ± 33.2	669.8 ± 35.9	679.3 ± 62.2
Dentate gyrus	419.0 ± 22.7	423.9 ± 22.6	468.9 ± 15.2	466.0 ± 44.4

Data are means ± SEM from the number (n) of mice indicated in parentheses. *Iba1* ionized calcium-binding adaptor molecule 1, *SSR* SSR240612, *WT* wild-type, *APP* amyloid precursor protein.

### Improvement of cerebrovascular function following B_1_R antagonism

#### Cerebral blood flow

**Figure 8:**
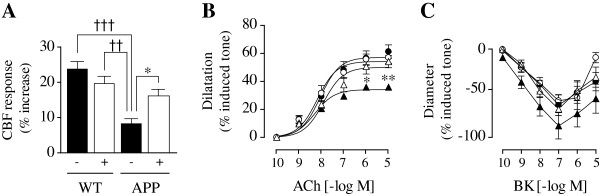
**SSR240612 improved evoked CBF responses to sensory stimulation *in vivo *and cerebrovascular reactivity *ex vivo*. (A)** In APP mice, B_1_R antagonism (+) significantly ameliorated, but did not fully restore, the cerebral blood flow (CBF) response evoked by whisker stimulation. **(B,C)** Isolated and pressurized middle cerebral artery segments from APP mice (▲) displayed impaired dilation to acetylcholine (ACh) **(B)** and reduced, albeit non-significantly, the biphasic response (contraction followed by relaxation) to bradykinin (BK) **(C)**. Treatment with SSR240612 in APP mice (Δ) completely rescued dilation to ACh (B) but had no significant effect on vascular responses to BK **(C)**. Error bars represent SEM. *P <0.05; **P <0.01, ***P <0.001, Two-way ANOVA followed by Newman-Keuls post hoc test. N = 4 to 6/group. (● WT; ○ WT treated). APP, amyloid precursor protein; B_1_R, bradykinin receptor 1; WT, wild-type.

The CBF response evoked by increased neuronal activity is impaired in APP mice [28,45], as found here in our two cohorts of adult mice compared to their respective WT controls (5 weeks: -65%, *P* <0.001; 10 weeks: -41%, *P* <0.05). SSR240612 treatment significantly ameliorated these sensory-evoked hemodynamic responses (+41% and +36%; respectively for 5 and 10 weeks, *P* <0.05), as illustrated in Figure 8A for the 5 weeks of treatment cohort. There was no added benefit with the longer treatment.

#### Cerebrovascular reactivity

In APP mice, the cerebrovascular contractile capacity is unaltered up to 21 months of age, whereas dilatory function is impaired early [28,31,46]. Hence, we tested whether SSR240612 could improve the dilatory deficits. As expected, arteries from adult APP mice featured significantly decreased dilatory responses to ACh over a wide range of concentrations, with a 40 to 50% reduction in EAmax compared to WT and no change in affinity, mean pD_2_ values being comparable (Figure 8B, Table 2). Following 5 or 10 weeks of SSR240612 administration, impaired cerebrovascular dilation to ACh in APP mice was completely normalized, with dose-dependent and maximal responses, as well as agonist potencies, being identical to those of WT (5 weeks of treatment illustrated in Figure 8B). The two treatment durations had similar efficacy and did not alter vascular reactivity to ACh in WT animals. To test whether long-term B_1_R blockade would influence the B_2_R-driven vasomotricity, cerebrovascular responses to BK were tested. BK elicited a biphasic response (contraction followed by relaxation) in all groups, with a tendency, although not significant, to constrict more and dilate less in APP mice (Figure 8C). SSR240612 had no effect on vascular responses to BK, despite a slightly larger, not significant, dilation in treated WT mice at the highest agonist concentration (Figure 8C, Table 2). Although beyond the scope of the present investigation, further experiments could involve testing the effects of selective B_2_R antagonists on the biphasic response to BK.

**Table 2:** Effects of SSR240612 treatments on cerebrovascular responses to ACh and BK

**SSR 5 weeks**	**WT (n = 4)**	**WT-SSR (n = 4)**	**APP (n = 4)**	**APP-SSR (n = 4)**
ACh (EA_max_)	57.2 ± 3.0	54.3 ± 2.5	34.2 ± 2.0 ††	50.1 ± 3.3**
ACh (pD_2_)	8.06 ± 0.2	8.19 ± 0.1	8.30 ± 0.2	7.91 ± 0.2
BK (max constr.)	−69.53 ± 13.2	−64.15 ± 7.3	−95.55 ± 16.4	−74.50 ± 8.3
BK (max dilat.)	−40.3 ± 11.4	−10.3 ± 6.4	−61.6 ± 18.1	−34.6 ± 12.6
**SSR 10 weeks**	**WT (n = 4)**	**WT-SSR (n = 5)**	**APP (n = 5)**	**APP-SSR (n = 5)**
ACh (EA_max_)	51.2 ± 2.7	67.8 ± 3.3	32.5 ± 1.9 †	49.2 ± 3.2 *
ACh (pD_2_)	8.03 ± 0.1	7.79 ± 0.1	8.37 ± 0.18	8.17 ± 0.2
BK (max constr.)	−54.11 ± 6.4	−77.67 ± 7.8	−70.77 ± 7.5	−63.78 ± 9.2
BK (max dilat.)	−23.5 ± 5.5	−8.6 ± 9.2	−44.9 ± 8.3	−31.3 ± 9.8

Data are means ± SEM from the number (n) of mice indicated in parentheses and expressed as the agonist maximal response (EA_max_) or affinity (pD2, -log[EC50]). EA_max_ (%) is the maximal dilation to ACh. For agonist response to BK, both the maximal constriction and dilatation from the induced tone (%) are indicated. Values for BK are negative, the zero value being the induced tone. ^†^*P* <0.05, ^††^*P* <0.01 when compared to non-treated WT controls; ^*^P <0.05, ^**^P <0.01 when compared to non-treated APP mice. *SSR* SSR240612, *ACh* acetylcholine, *BK* bradykinin, *WT* wild-type, *APP* amyloid precursor protein.

### SSR240612 effects on brain and vascular BK receptors

**Figure 9:**
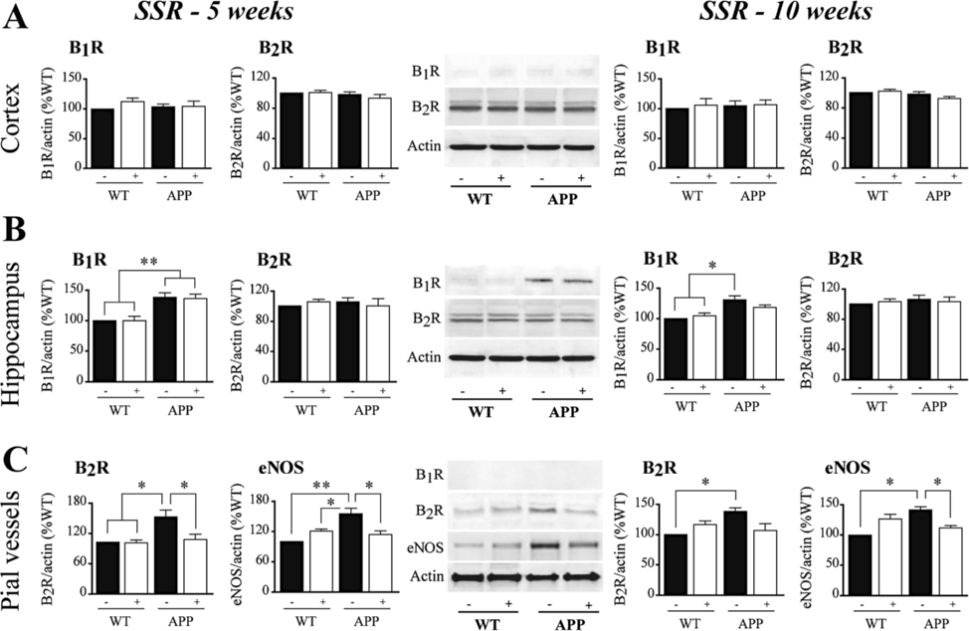
**Effects of SSR240612 treatment on cerebral kinin receptors and on endothelial NOS and vascular B**_**2**_**R protein levels. (A,B)** Single bands were detected at 37 and 135 kDa by anti-B_1_R and anti-eNOS antibodies, respectively, whereas anti-B_2_R antibodies decorated a characteristic doublet around 42 kDa. No change in cortical B_1_R and B_2_R **(A)** or in hippocampal B_2_R **(B)** protein levels was observed, but B_1_R were upregulated in the hippocampus of APP mice **(B)**, with no effect of treatment. **(C)** In pial vessels, protein levels of endothelial NOS (eNOS) and B_2_R were normalized by B_1_R blockade. Blots images illustrate the 5 weeks of treatment. *P <0.05; **P <0.01, Two-way ANOVA followed by Newman-Keuls post hoc test. Blots images illustrate the 5 weeks of treatment. N = 5 to 6/group. APP, amyloid precursor protein; B_2_R, bradykinin receptor 2.

The effects of SSR240612 on brain and vascular BK receptors were measured using western blot. B_1_R protein levels were significantly increased only in the hippocampus from APP mice, as shown in the two mouse cohorts treated for 5 or 10 weeks (+41%, *P* <0.01; and +32%, *P* <0.05; respectively) (Figure 9A,B). SSR240612 treatment slightly reduced this upregulation in hippocampus, bringing B_1_R protein levels in treated APP mice indistinguishable from those of WT controls after 10 weeks of drug delivery (Figure 9B, right). Neither genotype nor treatment altered B_2_R protein levels in cortex and hippocampus. Knowing that B_2_R and endothelial NOS (eNOS) activation mediate the effects of BK on brain arteries [33], their protein levels were measured in pial vessels. B_1_R protein was not detected in vascular extracts (Figure 9C), confirming our immunohistochemical observations. However, in APP mice, B_2_R and eNOS protein levels were increased in cerebral arteries (+40 to 50%; *P* <0.05), and both were normalized by SSR240612 administration independent of treatment duration (Figure 9C).

## Discussion

Our study i) shows a selective astrocytic upregulation of B_1_R, being almost exclusively associated with Aβ plaques, in the hippocampus of APP mice with impaired memory, and ii) demonstrates that chronic blockade of B_1_R significantly improves learning and memory performances, cerebrovascular function, as well as several anatomopathological AD hallmarks in APP mice with a fully developed pathology. These findings strongly support a deleterious effect of kinin B_1_R in AD pathogenesis.

### Selective B_1_R upregulation on Aβ plaque-associated astrocytes in hippocampus of APP mice

Previous *in vivo* studies in rats [17] or mice [18] showed increases in brain B_1_R binding sites or protein levels after a single i.c.v. infusion of human Aβ_1-40_. Here, with western blot analysis we found an upregulation of B_1_R protein levels in the hippocampus, but not cerebral cortex of approximately 11- to 12-month-old APP mice. Furthermore, using the cellular resolution of immunocytochemistry, we confirmed that B_1_R were upregulated in the hippocampus of APP mice, particularly in the hilus of the DG, a key segment of the entorhinal-hippocampal network for learning and memory that is impaired early by APP/Aβ overproduction [47]. Interestingly, recent evidence suggests that upregulation of B_1_R might occur in early stages of AD-associated neuroinflammation [2], particularly in the hippocampus where components of the KKS are activated in response to inflammatory stimuli [48].

Astrocytes and microglia are instrumental in the neuroinflammatory processes associated with AD pathogenesis [49], and activation of astrocytes and microglia can be evidenced in the brain of APP mice with their respective typical association outside and within Aβ plaques [50]. Yet, the prominent finding from double-immunofluorescence experiments was that upregulation of B_1_R was limited to astrocytes, mainly those located in the vicinity of Aβ deposits, that displayed characteristics of reactive astrocytes such as hypertrophic processes and soma, and upregulated GFAP immunostaining [51]. Our findings add to the previously reported upregulation of B_1_R on glial or neuronal cells in various pathologies, including in the brain of epileptic patients [52] and spinal cord of diabetic rats [53].

In contrast to a recent report of B_1_R upregulation in brain microvessels of Tg-SwDI mice, published online while our work was under review [54], we did not detect cerebrovascular B_1_R expression in our 12-month-old APP mice. Beyond the use of different anti-B_1_R primary antibodies, this apparent discrepancy is likely due to the robust deposition of cerebrovascular amyloid (CAA) in Tg-SwDI mice [55]. Indeed, J20 APP mice at the age used in our study are literally free of CAA [30]. Together, these findings reinforce our conclusion that astroglial B_1_R upregulation is associated primarily, if not exclusively, with aggregated Aβ species.

### SSR240612 and recovery of cognitive deficits in APP mice

We found that chronic B_1_R antagonism improved spatial learning and memory in APP mice, abilities that depend largely on hippocampal integrity [56]. Moreover, prolonged B_1_R blockade enhanced the baseline levels of the memory-related Egr-1 protein in the DG, a brain region previously associated in APP mice with cognitive deficits, reduced immediate-early gene expression and altered synaptic activity [57,58]. Although further experiments would be required to confirm this hypothesis, it is conceivable that under chronic B_1_R blockade with SSR240612 in APP mice, BK can then act on its normal target and preferentially activate constitutive B_2_R that are considered neuroprotective and able to prevent memory loss [59].

**Figure 10:**
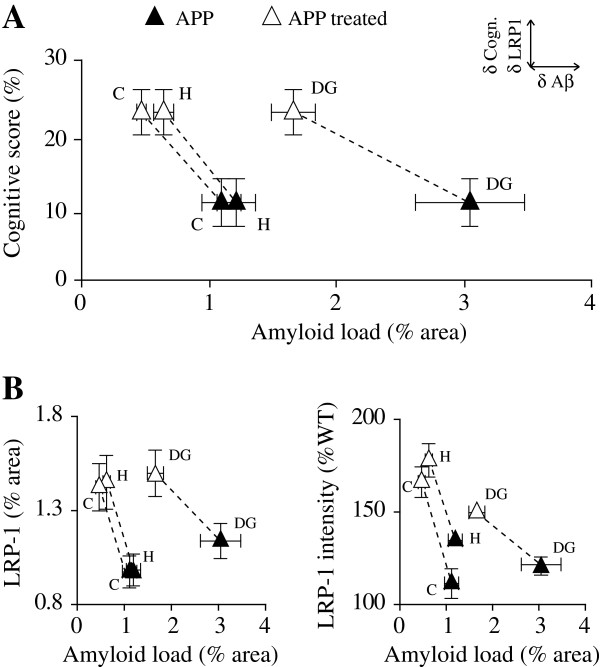
**Relationships between cognitive performances, amyloidosis and LRP-1 expression in APP mouse brain. (A)** Relationship between Aβ plaque load and cognitive score (see Material and Methods). **(B)** Relationship between Aβ plaque load and LRP-1 immunoreactivity (left, % area; right, intensity). Illustration for the 10-week treatment, but similar relationships were obtained after the 5-week treatment. Error bars represent SEM. N = 5/group (▲ APP; Δ APP treated). Aβ, amyloid-beta; APP, amyloid precursor protein; C, cortex; H, hippocampus; DG, dentate gyrus; LRP-1, lipoprotein receptor-related protein.

The findings of decreased soluble Aβ_1-42_ and Aβ plaque load in both cortex and hippocampus after SSR240612 treatment suggested that B_1_R blockade interfered with the amyloidogenic cascade and, particularly, with the deposition of the fast aggregating Aβ_1-42_ species. Interestingly, long-term B_1_R blockade in APP mice resulted in increased production of MMP9 that is known to promote non-amyloidogenic processing of APP [60], and in increased cerebrovascular LRP-1 immunoreactivity, a vascular mediator of Aβ efflux from brain to blood [61]. Our results may thus suggest a stimulated Aβ clearance at the BBB following B_1_R blockade. To date, both soluble [62,63] and insoluble Aβ levels have been incriminated in cerebrovascular and cognitive deficits observed in APP mice [64,65], but there is also strong evidence for no relationship between the Aβ pathology and cognitive performance in APP mice [30,66,67]. We cannot ascertain whether or not the normalized memory performance in APP mice after B_1_R blockade is related to the concurrent decreases in soluble Aβ_1-42_ species and Aβ plaque load. However, the relationships between high Aβ plaque load and low cognitive scores - particularly striking in the DG - and of high Aβ plaque load and low LRP-1 immunostaining (Figure 10) were shifted toward low plaque load and high cognitive performance, and toward low plaque load and high LRP-1 following SSR240612 treatment, suggesting a negative impact of Aβ on cognition possibly due to its lack of clearance from brain.

### SSR240612 effects on reactive astrocytes and microglia

Aβ is the main initiating factor of the inflammatory cascade in APP mice, and specific Aβ domains can stimulate the KKS [68], and it has been argued that brain injury in AD is primarily caused by Aβ-induced neuroinflammation [69]. Interesting to the present study was that B_1_R antagonism selectively reduced microglial activation, and most strikingly reactive microglia associated with Aβ plaques. Suggestive of microglial migration and aggregation around plaques, we show that despite an unchanged total microglial cells number in APP mice brain, the intensity of anti-microglia staining is increased in APP compared to WT controls, and significantly reduced following B_1_R blockade. It thus seems that the reduction in Aβ plaque load contributed to the silencing of the reactive microglial cells. This would be consistent with Aβ-induced migration of phagocytic microglia, and with plaque-associated reactive microglia contributing to the inflammatory response and exhibiting a neurotoxic phenotype [70,71]. Intense and patchy GFAP-immunostained astrocytes persisted after SSR240612 treatment, suggesting that astrocytes have maintained a reactive phenotype, as supported by the enduring increases in B_1_R protein levels in activated astrocytes in APP mouse hippocampus (Figure 2). This might indicate a compensatory astroglial activation in response to the reduction in microgliosis [72], possibly related to the ability of reactive astrocytes to attenuate microglia-derived neurotoxicity [44].

### Improvement of cerebrovascular function following B_1_R blockade

A remarkable outcome from the present study was the normalization of CBF responses and cerebrovascular reactivity in adult APP mice by chronic B_1_R antagonism, irrespective of the treatment duration. Growing evidence supports cerebrovascular impairment as an early event of AD pathogenesis [1]. Since B_1_R blockade failed to reduce soluble Aβ_1–40_ in APP mice, commonly perceived as a seed for Aβ_1–42_ deposition in brain vessels leading to CAA [73], recovery of cerebrovascular reactivity in APP mice likely happened through reduction of soluble Aβ_1–42_ since they display virtually no CAA at this age [30]. Aβ_1–42_ is detrimental to cerebrovascular function [45] through increased oxidative stress [31,74] and inflammation [75,76]. Hence, cerebrovascular recovery likely resulted, at least in part, from the ability of SSR240612 to antagonize the B_1_R-mediated production of vascular NADPH oxidase-derived reactive oxygen species [10] and inflammatory cytokines [77], as recently documented in aorta from a rat model of insulin resistance. In treated APP mice, SSR240612 normalized the increased levels of B_2_R and eNOS, two key proteins in endothelial-mediated vasomotor responses, which are known to be upregulated by oxidative stress and inflammation [8]. Hence, the high eNOS protein levels in APP mice may reflect a compensatory upregulation in response to the reduced NO bioavailability following its trapping by reactive oxygen species [31,74], as also reported in a model of diabetes-associated vascular disease [78].

## Conclusions

Our findings indicate that upregulation of B_1_R in APP mice results in deleterious effects on the cerebral vasculature and brain parenchyma. They further demonstrate the capacity of the B_1_R antagonist SSR240612 to abrogate amyloidosis, cerebrovascular and memory deficits. These observations provide support for a neuroprotective role for B_1_R antagonism in AD, pointing to the need to better understand the role of the KKS in AD pathogenesis.

## Abbreviations

5-HT: 5-hydroxytryptamine (serotonin); Aβ: Amyloid-beta; ACh: Acetylcholine; AD: Alzheimer’s disease; APP: Amyloid precursor protein; B1R: Bradykinin receptor 1; BACE: Beta-site APP-cleaving enzyme (β-secretase); BBB: Blood–brain barrier; BK: Bradykinin; CAA: Cerebrovascular amyloid; CBF: Cerebral blood flow; CD11b: Cluster of differentiation molecule 11b; CSF: Cerebrospinal fluid; DG: Dentate gyrus; DMSO: Dimethyl sulfoxide; EAmax: Maximal response; Egr-1: Early growth response protein 1; ELISA: Enzyme-linked immunosorbent assay; eNOS: Endothelial nitric oxide synthase; GFAP: Glial fibrillary acidic protein; Iba1: Ionized calcium-binding adaptor molecule 1; i.c.v.: Intracerebroventricular; IHC: Immunohisto chemistry; KKS: Kallikrein-kinin system; KO: Knockout; LRP-1: Lipoprotein receptor-related protein 1; MCA: Middle cerebral artery; MMP9: Matrix metallopeptidase 9; PDGF: Platelet-derived growth factor; PFA: Paraformaldehyde; RT: Room temperature; SEM: Standard error of the mean; WB: Western blot; WT: Wild-type.

## Competing interests

The authors declare that they have no competing interests.

## Authors’ contributions

BL designed the study and performed all experiments and statistical analyses - except for CBF measurements of the 5-week treatment cohort - and drafted the manuscript (text and figures). XKT performed the CBF measurements for the 5-week treatment cohort and provided technical advises at all steps of the study. KL developed and characterized the primary anti-B_1_R and -B_2_R antibodies under RC’s supervision. RC co-supervised BL’s work, participated in the design of the study and helped draft the manuscript. EH designed and supervised all steps of the study, and corrected the manuscript. All authors read and approved the final manuscript.
